# Influence of PLIN5 and lipid composition on lipid droplet contact sites with other organelles

**DOI:** 10.1016/j.bbrep.2025.102402

**Published:** 2025-12-08

**Authors:** Mahsa Mohammadian, Shima Asfia, Ralf Seemann

**Affiliations:** Department of Experimental Physics and Center for Biophysics, Saarland University, Saarbrücken, Germany

**Keywords:** Lipid droplet, Large unilamellar vesicle, Contact site, Lipidic bridge, Protein tether, Perilipin 5, PLIN5

## Abstract

Lipid droplets (LDs) maintain cellular lipid homeostasis through dynamic interactions with other organelles. Understanding how these contact sites form is crucial for uncovering the mechanisms of lipid exchange and signaling. In this study, we used an in vitro model to investigate how lipid composition and the LD-associated protein perilipin 5 (PLIN5) influence contact formation between an LD monolayer and a bilayer membrane. Artificial LDs consisting of triolein and coated with either a DOPE or DOPC monolayer containing PLIN5 or not were incubated with large unilamellar vesicles (LUVs) that mimic the bilayer membrane of the organelle. Using double fluorescence labeling of the LUV bilayer and the core, we can distinguish between fusion of the LUV bilayer with the LDs and stable attachment of LUVs to the LD’s surface. Our results show that the probability of fusion between LDs and LUVs is greatly increased for DOPE-coated LDs, while PLIN5 promotes the stable attachment of LUVs to the LD’s surface and prevents fusion. These observations illustrate how certain lipid and protein components can modulate contact formation between LDs and membranes in a controlled in vitro system, and provide a basis for future studies on the molecular mechanisms of organelle communication.

## Highlights

• Lipid droplets interact with other organelles by forming contact sites.
• Lipidic bridges form transiently while protein tethers are more stable.
• PLIN5 keeps LUVs at distance and promotes stable protein tethers.
• Lipid composition and PLIN5 regulate LD-organelle interaction modes.
• DOPE-coated LDs show higher lipid fusion than DOPC-coated LDs.

## 1 Introduction

**Fig. 1:**
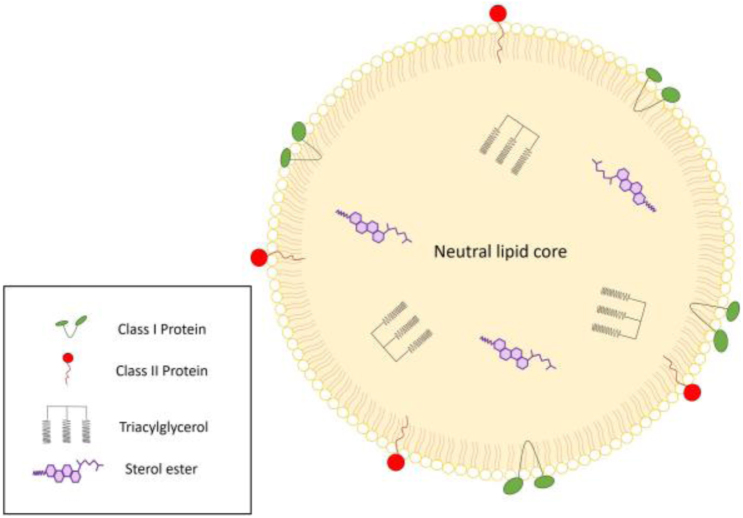
Schematic of a LD including various protein classes embedded in the phospholipid monolayer decorating the LD.

Lipid droplets (LDs) are known to serve as reservoirs for neutral lipids and play an important role in the control of energy and lipid metabolic processes, membrane biogenesis and the synthesis of signaling molecules. LDs consist of the neutral lipids in their core, primarily triacylglycerol (TAG) and sterol esters (SE), which are surrounded by a phospholipid monolayer with different proteins decorating the surface [1], [2], see Fig. 1. To fulfill their crucial function, lipid droplets are known to interact extensively with various cellular organelles [3], [4]. One of the most significant interaction sites is the endoplasmic reticulum (ER), which serves as a key platform for LD formation, expansion, and budding. Another important organelle in this network is the mitochondrion, where LDs supply fatty acids derived from stored neutral lipids for oxidation and energy production [5]. Additionally, peroxisomes establish specialized contact sites with LDs, known as pexopodia [6]. These structures enable peroxisomal protrusions, lined by the inner bilayer leaflet of the peroxisome, to extend into the lipid droplet core. Notably, pexopodia are enriched with components involved in β oxidation, highlighting their potential role in lipid metabolism [7].

**Fig. 2:**
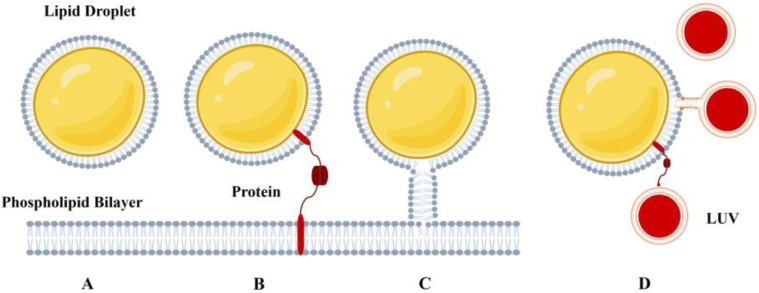
Schematic of possible ways of LD contact sides with and without protein connection. (A) LDs and Phospholipid bilayer in close proximity (B) Protein tethers connecting LDs with phospholipid bilayer (C) Lipidic bridge connecting LDs with phospholipid bilayer (D) in vitro method to model LDs contact sites with LUVs.

When two organelles are in close proximity (typically 10–70 nm apart) and physically linked, they form contact sites that enable the efficient and rapid exchange of different molecules through active or passive transport [1], [5], Fig. 2. The formed contact sites are typically protein-mediated structures that are known as “protein tethers” [8]. According to the literature, the word “tether” has been used to define a protein linkage between the LDs and other organelles [9], [10]. These protein tethers play an important role in keeping the interacting organelles at a specific distance, thereby establishing the framework for the architecture of the contact site (Fig. 2) [1]. These contact sites contain various proteins, categorized as either “effectors”, which carry out specific functions like material transfer, or “regulators”, which modulate the contact site based on the cell’s functional state [1], [8].

Although protein tethers are the main topic in the field of contact site investigation, also lipidic structures forming connections between LDs and the endoplasmic reticulum (ER), and in some cases with other organelles, have been frequently observed [11], [12]. These findings have drawn attention to the possible involvement of lipidic bridges in facilitating the formation of LD contact sites [3]. Lipidic bridges can serve as channels connecting the phospholipid membrane of LDs to the outer leaflet of the phospholipid bilayer of the ER (Fig. 2). These architectural features have been documented across various cell types and have been reported to establish connections between nearly all intracellular LDs and the ER in yeast [13]. Since these lipidic bridges may not be rigid enough for inter-organelle contacts, they probably coexist with, rather than replacing, protein-based tethers, which are essential for maintaining structural integrity of these contact sites [1]. Studies have shown that similar lipidic bridges have been seen mostly in the LD–Peroxisome connections and LD–ER bridges, which can form de novo, reconnecting LDs that were previously detached from the ER. This suggests that these bridges have a more active role than just being leftovers from the LD biogenesis process [7], [14], [15].

In mammalian cells, the predominant component of the phospholipid monolayer covering an LD is phosphatidylcholine (DOPC), which can make up to 60% of the total membrane composition. After DOPC, the next most common phospholipid is phosphatidylethanolamine (DOPE) and, with smaller amounts, phosphatidylinositol (DOPI), phosphatidylserine (DOPS), and sphingomyelin (SM) [16]. The phospholipid monolayer of a LD is enriched with specific proteins [1], [2] that facilitate various cellular processes. So far, it has been shown that there are different types and classes of proteins decorating LDs and two of these classes have been broadly studied. Class I proteins, which possess hydrophobic hairpin motifs are thought to localize into LDs from the ER during their formation. Class II proteins access the LD’s surface directly from the cytosol through amphipathic helices or via multiple amphipathic and hydrophobic helices [17], [18].

There are several proteins contained in the LD coating that are associated with the connection of LDs with different organelles. Among these, Seipin and Perilipin 5 (PLIN5) (one of the known proteins from PLIN family protein) are both well-conserved transmembrane proteins, which show their importance in the cellular energy homeostasis [3], [19]. Seipin, a transmembrane protein, localizes to ER–LD contact sites and promotes the formation of these contacts [19]. PLIN5, a class II protein is known for the LD–mitochondria connection and recognized as a LD targeting protein [20]. It has been reported that PLIN5 forms contacts with phospholipid membranes in cells [10], [21], with one possible explanation for the observed contacts being a direct interaction between PLIN5 and the phospholipids of the LUV bilayer [9]. However, the molecular mechanism underlying PLIN5-mediated tethering to organelle membranes remains to be established.

PLIN5, on one hand, helps to stabilize lipid droplets, preventing their coalescence and fusion and is involved in the storage of lipids within the LDs [22], [23]. On the other hand, PLIN5 helps to keep triglycerides and other neutral lipids safely stored inside the lipid droplet, protecting them from degradation [24]. Besides, PLIN5 plays a role in regulating lipolysis, the process by which stored triglycerides are broken down into fatty acids and released into the cell for energy production. It can either promote or inhibit lipolysis depending on the cellular context and signaling pathways [25]. Moreover, PLIN5 in addition to having a potential role in LD budding, can interact with other proteins involved in lipid metabolism and intracellular signaling pathways [26], [27]. These proteins can interact with other proteins of the PLIN family, such as PLIN1 and PLIN2 to regulate LD function collectively [26], [28].

Studies have shown that mutation and dysfunction of LD proteins such as perilipin family members and seipin can be associated with several human diseases, including obesity, diabetes, steatohepatitis and lipodystrophy [29], [30], [31]. An in-depth understanding of the functionality of the LD contact site network holds significant promise in shedding light on the underlying mechanisms of lipid-associated pathological conditions [32], [33].

In this study, LDs coated with either a DOPC or DOPE monolayer, containing PLIN5 or not, were brought into contact with Large Unilamellar Vesicles (LUVs) that are comparable in size to cellular organelles such as mitochondria. The LUVs contain a phospholipid dye (rhodamine) in their outer membrane and a water-soluble dye (Cy5 labeled dextran) in their core (Fig. 2). After incubation, the mixture was observed in a microfluidic channel with a fluorescence microscope. Due to the double fluorescent labeling we could distinguish between the fusion of the LUV bilayer with the LDs and stable attachment of LUVs to the LD’s surface. Our findings reveal that LDs coated with DOPC or DOPE tend to fuse with LUVs, with fusion occurring at a substantially higher probability for DOPE-coated LDs. When adding PLIN5 to the phospholipid LD coating, LUVs were found in stable attachment to the LDs, despite no proteins were present in the LUVs; a situation referred to as a protein tether in the literature, although the exact molecular mechanism is unknown [21].

## 2 Results and discussion

### 2.1 Lipidic bridge in LD’s contact sites with LUVs

**Fig. 3:**
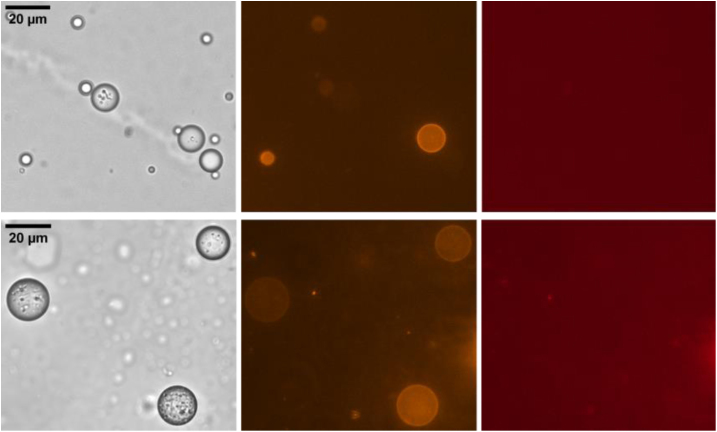
Fusion behavior of LD’s monolayer with LUVs after the incubation time. LD’s monolayer consists of DOPC (top row) and DOPE (bottom row). The different columns show the same spot of one sample with different microscopy contrast methods, respectively fluorescent wavelengths. (left) Bright field micrographs, (middle) rhodamine dye channel and (right) Cy5 dye channel. Due to the fairly weak fluorescence signal, the brightness of the images was increased to the noise level to demonstrate that no LD–fluorescence signal is hidden in the background.

Fig. 3 shows results of the first set of experiments where DOPC or DOPE covered LDs were brought in contact with LUVs composed of DOPC and DOPE with a molar ratio of 60:40 (further examples can be found in the Supplementary data, Figs. S1 and S2). After the incubation time of 60 min, a certain fraction of the initially invisible LDs turned orange showing a rhodamine fluorescent signal. A parallel Cy5 fluorescent signal from the core of the LUVs could not be observed. This indicates that lipidic bridges between LDs and LUVs were formed and rhodamine–PE was transferred into the LD’s monolayer. In our experiments, it remains unclear whether the LUVs fully fused with the LDs, completely donating their phospholipid bilayer to the LD monolayer, or if they only made short contact, exchanged some phospholipids, and then detached. However, the lack of Cy5 signal on the surface of the LDs clearly indicates that the LUVs do not remain attached, suggesting that the formed lipidic bridges are transient.

**Fig. 4:**
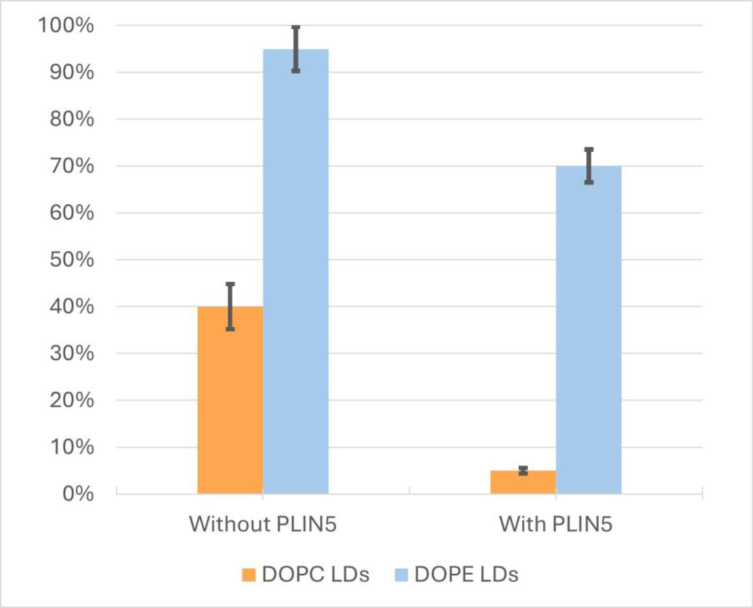
Probability of LD and LUV fusion as function of the LD’s monolayer. LDs coated with DOPC (orange) and DOPE (blue) are shown on the left, and LDs coated with DOPC (orange) and DOPE (blue) with PLIN5 are shown on the right. Quantification was performed by counting a minimum of 150 LDs per condition across repeated independent experiments. The error bars ±(1–5) % reflect the detection accuracy.

To evaluate how lipid composition affects the formation of lipidic bridges, defined here as the transfer of rhodamine–PE from LUVs to the LD monolayer, we compared LDs coated with DOPC and DOPE monolayers. These statistics are based on the minimum number of 150 LDs per experimental condition across multiple independent experiments. After 60 min of incubation, only about 40% of the DOPC-coated LDs displayed rhodamine fluorescence, indicating phospholipid uptake from the LUVs. In contrast, approximately 95% of DOPE-coated LDs showed strong rhodamine signals, suggesting a significantly higher fusion efficiency, see Fig. 4. This difference in fusion probability of the LDs aligns with the larger surface tension of a DOPE covered triolein–water interface with respect to a DOPC covered triolein–water interface of ≈2.3mN/m and 1.5mN/m, respectively, and the higher diffusion rate of DOPE phospholipids in comparison with DOPC phospholipids of $\approx 8\;\mu m^{2}/s$ and $18\;\mu m^{2}/s$, respectively [34], [35].

### 2.2 Role of PLIN5 in LD contact sites

**Fig. 5:**
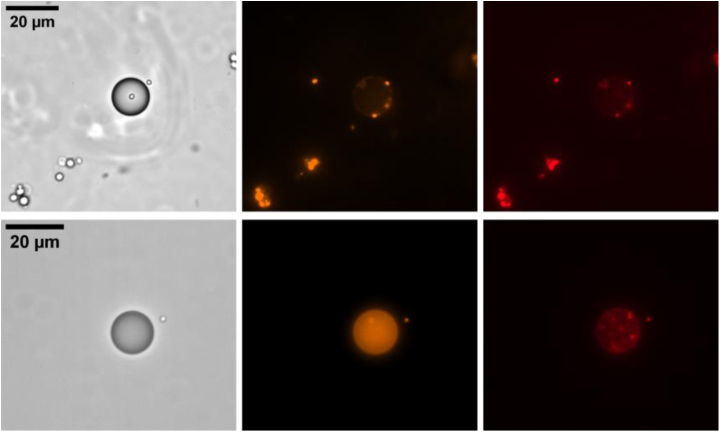
Fusion behavior of PLIN5-decorated LDs with LUVs after the incubation time. LD’s monolayer consists of DOPC (top row) and DOPE (bottom row), plus PLIN5. Different columns show bright field images (left), rhodamine dye channel (middle) and Cy5 channel (right). The different columns show the same spot of one sample with different microscopy contrast methods, respectively fluorescent wavelengths.

To additionally investigate the role of PLIN5 proteins in forming contact sites between LDs and LUVs, a similar set of experiments was conducted but with PLIN5 contained in the phospholipid monolayer covering the LDs. Similarly to the previous experiments, we also find LDs that show a fairly homogeneous rhodamine signal after the incubation time, Fig. 5 (Further examples can be found in the Supplementary data, Figs. S3 and S4). But the probability of finding LDs with a homogeneous rhodamine signal dropped to 5%, and 70%, respectively. In the presence of PLIN5, the total probability of phospholipid uptake from LUVs decreased by factors of 1.3 for DOPE-coated LDs and 8 for DOPC-coated LDs, respectively.

In this set of experiments with PLIN5, in comparison to the previous condition without PLIN5, we additionally find LDs with distinct individual fluorescence spots with both rhodamine and Cy5 signal, which are not visible in the bright field micrographs. These bright spots can thus be identified as LUVs that remain attached to the LDs by so-called protein tethers without undergoing fusion. While in previous observations lipidic bridges formed only transiently, protein tethers remained stable over a longer period of time.

The qualitative observation of these LUVs that are attached to the LDs, together with the quantitative observation of a smaller percentage of LDs that fused with LUVs suggests that PLIN5 stabilizes LUV–LD contacts without promoting fusion, or even prevents fusion of LUVs with LDs. This observation strongly supports the interpretation of PLIN5-mediated interactions at LD–LUV contact sites, known as protein tethers (see Fig. 5) that keep the LUVs at a certain distance and thus rather prohibit unspecific fusion.

## 3 Conclusion and outlook

In this study, we introduced a model system to investigate the contact sites of LDs and LUVs, representing the interaction of LDs with the bilayer membrane of cell organelles. To test the effect of the LD’s membrane, respectively, phospholipid composition, we conducted experiments with two different phospholipid compositions (DOPC and DOPE) with and without additional PLIN5.

While DOPE enhances the fusion probability of LDs with LUVs, presumably due to the formation of transient lipidic bridges, we observed that this behavior changes considerably when PLIN5 is incorporated into the LD monolayer. In the presence of PLIN5, LUVs remain stably attached to the LD’s surface, and the fusion probability of DOPE-coated LDs is reduced by about 8 times. This observation can be explained as a result of the formation of protein tethers that keep the LUVs at a fixed distance from LDs. Our results therefore support a direct interaction between PLIN5 and the phospholipids of the bilayers, which is also suggested by Thiam et al. [9].

The enhanced fusion observed for DOPE-coated LDs compared to DOPC can be attributed to the higher lipid mobility and lower packing density of DOPE [36]. These properties result from the truncated-conical geometry of DOPE versus the cylindrical geometry of DOPC [34], [36]. Their impact on the fusion probability emphasizes the significance of the biophysical properties of lipid monolayers in mediating inter-organellar communication and structural interactions.

Our findings thus support the idea that PLIN5 plays a regulatory role in forming functional connections between LDs and other organelles, such as mitochondria, potentially influencing lipid metabolism and energy homeostasis. Although the molecular interactions between these protein tethers and their opposing partners remain unknown, we can state that the interaction of PLIN5 with the organelle membrane can be established by the phospholipids and without specific proteins. These insights advance our understanding of the mechanisms regulating LD interactions and provide a foundation for future research into the dynamic regulation of organelle communication. Finally, understanding how these molecular interactions contribute to broader cellular processes, such as lipid metabolism, energy homeostasis, and signal transduction, could pave the way for therapeutic interventions targeting metabolic disorders and related diseases. Future studies could extend this work by exploring the effects of mutant forms of PLIN5 or alternative proteins on LD contact site formation, which would further reveal the mechanisms of protein-mediated tethering and fusion. In addition to the protein-phospholipid interaction observed in this study, this platform could also be used to investigate how PLIN5 interacts with different types of phospholipids.

## 4 Materials and methods

### 4.1 Molecules

1,2-Dioleoyl-sn-glycero-3-phosphocholine (DOPC), 1,2-dioleoyl-sn-glycero-3-phospho-ethanolamine (DOPE) and 1,2-dioleoyl-snglycero-3-phosphoethanolamine-N-(lissamine rhodamine B sulfonyl) (rhodamine–PE) were acquired from Avanti Polar Lipids and Cy5 labeled dextran was purchased from Creative Biolabs. Glyceryl trioleate (triolein, T7140) and squalene (S3626) were purchased from Sigma-Aldrich. Recombinant Bovine Perilipin-5 (PLIN5) were ordered from Cusabio Technology LLC. For microfluidic device preparation, Sylgard 184 Silicone Elastomer Kit (PDMS) was obtained from Dow Corning. In this manuscript, buffer solution refers to 150 mM potassium chloride (KCl) purchased from Sigma-Aldrich. All the phospholipid stocks were dissolved in chloroform and stored at $-20{\;}^{\circ }$C.

### 4.2 3D-chip fabrication

The layout of the microfluidic structure was designed using AutoCAD software. The design consisted of one straight channel with dimensions 15 mm × 1 mm × 100 μm (length × width × height). The positive mold of this structure was fabricated by standard contact photolithography using the negative photoresist SU-8 on 2 inch Si-wafer. Sylgard 184 was prepared following the instructions of the manufacturer including a thorough mixing and degassing period of 15 min. Subsequently, the degassed PDMS mixture was poured directly onto the SU-8 photoresist master and cured at a temperature of 100 ${}^{\circ }$C for a duration of 2 h. Upon completion of the curing process, the cured PDMS material was separated from the SU-8 mold and inlet and outlet holes were punched into the PDMS at the two ends of the bottom channels. The thus prepared PDMS microchip was sealed with a glass cover slip using plasma bonding (Diener Electronics). The assembled microfluidic device was heated at 95 ${}^{\circ }$C for 1 h to increase the bonding strength between PDMS and glass.

### 4.3 Large unilamellar vesicle (LUV) preparation

Large unilamellar vesicles (LUVs) were prepared using DOPC and DOPE in a 60:40 molar ratio, incorporating two fluorescent dyes (Rhod-PE and Dextran-Cy5) at 2% of the total phospholipid concentration, to facilitate monitoring the vesicles and distinguishing different interaction pathways with LDs. Rhodamine–PE is a fluorescent-labeled phospholipid and Dextran-Cy5 is a fluorescent-labeled sugar, which enable them to locate on the surface and in the core of LUVs, respectively. Lipids and fluorescence dyes were mixed with the previously mentioned molar ratios and with a total concentration of 1 mg/mL in chloroform and vacuum dried for about one hour to ensure complete evaporation of the solvent. The dried lipid powders were re-suspended in 150 mM KCl buffer by pipetting and vortexing. Using an Avanti mini extruder, the suspension was subsequently passed 10 times through a 1 μm polycarbonate filter (Avanti) at $50{\;}^{\circ }$C, according to the company’s instructions. To optimize the number of vesicles for each experiment, serial dilutions of LUVs were prepared and tested prior to the experiments. A 1:100 dilution of the original solution showed better visibility of the LUVs with minimal background noise and resulted in a final concentration of $8.67\times 10^{8}$ vesicles per mL of KCl buffer.

### 4.4 PLIN5 reconstitution

Recombinant PLIN5 protein was purchased from Cusabio Technology LLC, with a reported purity of 94% according to the manufacturer’s certificate of analysis. Upon receipt, the lyophilized powder was briefly centrifuged to collect the material at the bottom of the vial and reconstituted in DI water to a final concentration of 1 mg/mL, following the manufacturer’s instructions. For long-term storage, glycerol was added to a final concentration of 30%, and the protein solution was aliquoted and stored at −20°C or −80°C [37]. The reconstituted PLIN5 protein was incorporated into the phospholipid monolayer for some experimental conditions.

### 4.5 Lipid droplet (LD) preparation

For the preparation of the lipid droplets, a chloroform droplet containing 2 w% of phospholipid (DOPC or DOPE phospholipid based on the experiment) was dried in a glass falcon under vacuum for 60 min to fully evaporate the existing chloroform in the sample. To the dried phospholipids, 250 μL triolein oil and for some cases, where PLIN5 was tested in the experiments, 4 μL of 0.1 mg/mL protein solution (prepared as described above). In the next step, the mixture was diluted with (8–10) mL KCl buffer, then a solution of lipid–oil droplets in buffer was formed by mixing the suspension with a magnetic stirrer for about 5 min at 250 rpm. The size of lipid droplets was controlled by the intensity and the speed of the stirrer; longer and faster stirring resulted in smaller sizes of LDs. To be able study the LDs individually and more conveniently under our fluorescence microscope, the mentioned time and speed were chosen that resulted in LDs with a size range of 5μm–20 μm. To observe the lipid droplets in contact with LUVs under the fluorescence microscope, 10 μL of the LUV solution was added to 60 μL of LDs dispersion in a vial (6:1 volume ratio) and incubated for 60 min. Afterwards, the LD–LUV solution was injected by pipetting into the microchip channel and observed by optical fluorescence microscopy. The experiments were conducted at low LD density so that LD coalescence events could be safely neglected, allowing for straight forward statistical interpretation of the observed LD–LUV interaction.

### 4.6 Inverted fluorescence microscopy

Fluorescence microscopy images were acquired with an inverted AXIO Observer 7 microscope (Carl Zeiss Microscopy GmbH) equipped with a fluorescence LED (Colibri7) and a CMOS camera (Axiocam 712). The fluorescence imaging was conducted using the filter excitation wavelengths of 538–562 nm (for Rhodamine) and 614–647 nm (for Cy5) and the filter emission wavelengths of 570–640 nm and 659–759 nm, respectively. The images were obtained with a 40× objective (NA: 0.95) and 150 ms exposure time. It is worth mentioning that all experiments were performed at a room temperature of $23{\;}^{\circ }$C.

## CRediT authorship contribution statement

**Mahsa Mohammadian:** Writing – original draft, Software, Investigation, Formal analysis. **Shima Asfia:** Writing – original draft, Software, Investigation, Formal analysis. **Ralf Seemann:** Writing – review & editing, Supervision, Funding acquisition, Conceptualization.

## Declaration of competing interest

The authors declare the following financial interests/personal relationships which may be considered as potential competing interests: Ralf Seemann reports financial support was provided by German Research Foundation. If there are other authors, they declare that they have no known competing financial interests or personal relationships that could have appeared to influence the work reported in this paper.

## Data Availability

Data will be made available upon request.

## Appendix A Supplementary data

MMC S1 Supplementary infomation, additional fluorescence images of LD-LUV interactions.
