# Supplementary material for: Influence of PLIN5 and lipid composition on lipid droplet contact sites with other organelles
Source: Biochem Biophys Rep. 2025 Dec 8;45:102402. doi: 10.1016/j.bbrep.2025.102402 (PMC12743417; doi:10.1016/j.bbrep.2025.102402)
Supplement: MMC S1 — Supplementary infomation, additional fluorescence images of LD-LUV interactions. [file mmc1.pdf]

Supporting Data to the Manuscript:

Influence of PLIN5 and Lipid Composition on Lipid Droplet Contact Sites with other Organelles

Mahsa Mohammadian[1,‡], Shima Asfia[1,‡], Ralf Seemann[1,*]

November 25, 2025

Department of Experimental Physics and Center for Biophysics,
Saarland University, Saarbrücken, Germany
‡ Authors contributed equally
* Correspondance to: r.seemann@physik.uni-saarland.de

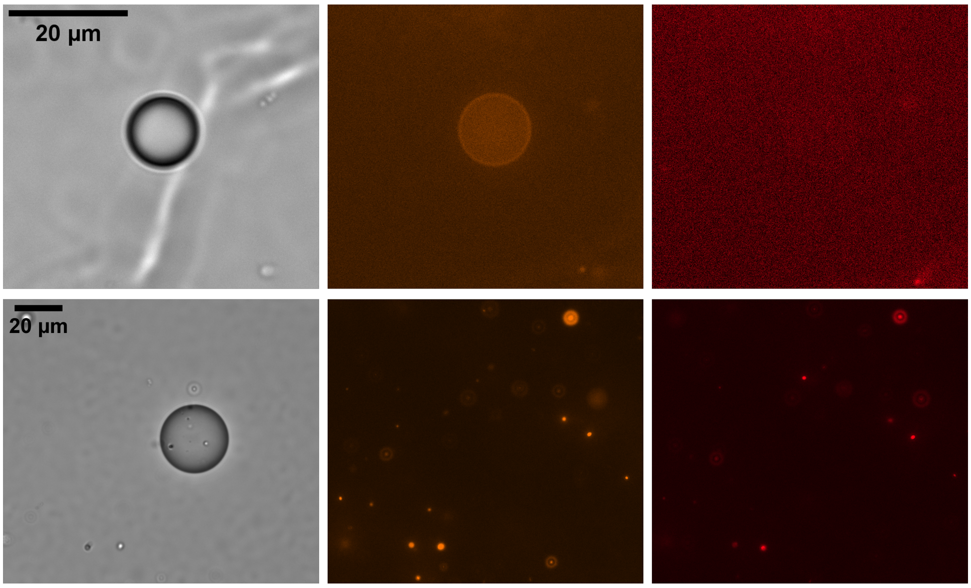

Figure S1: Additional representative images with the same experimental condition as those shown in Fig. 3 (top row) of the main text: Fusion behavior of LD's monolayer with LUVs after the incubation time. LD's monolayer consists of DOPC. The different columns show the same spot of one sample with different microscopy contrast methods, respectively fluorescent wavelengths. (left) Bright field, (middle) rhodamine dye and (right) Cy5 dye.

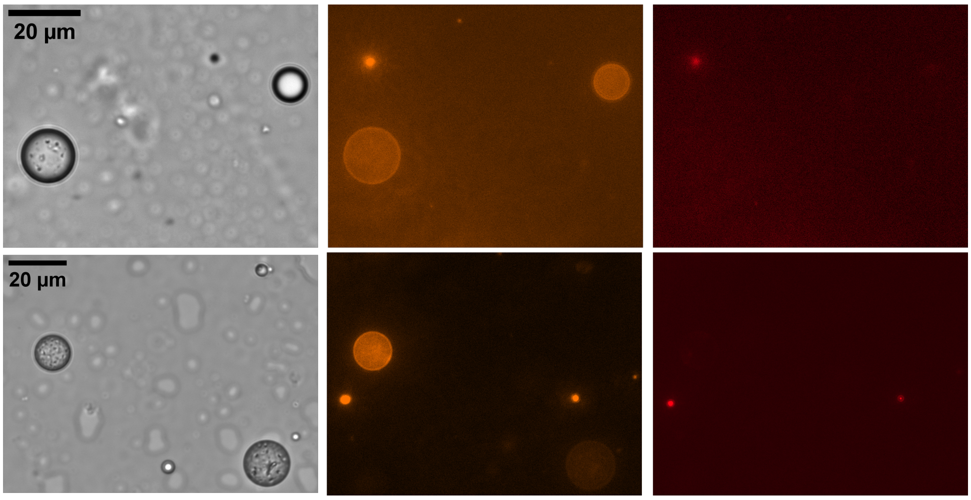

Figure S2: Additional representative images with the same experimental condition as those shown in Fig. 3 (bottom row) of the main text: Fusion behavior of LD's monolayer with LUVs after the incubation time. LD's monolayer consists of DOPE. The different columns show the same spot of one sample with different microscopy contrast methods, respectively fluorescent wavelengths. (left) Bright field, (middle) rhodamine dye, and (right) Cy5 dye.

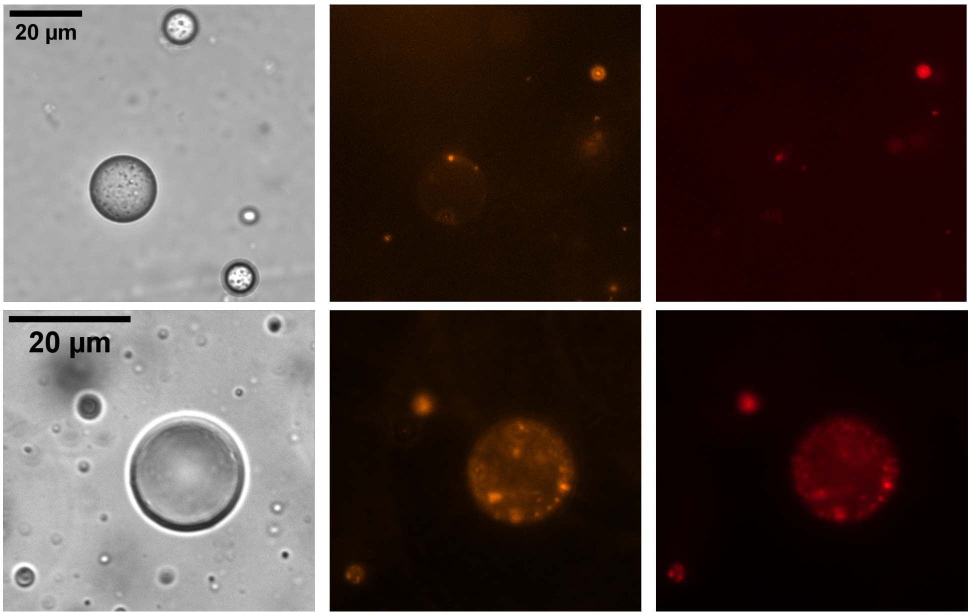

Figure S3: Additional representative images with the same experimental condition as those shown in Fig. 5 (top row) of the main text: Fusion behavior of DOPC and PLIN5 decorated LDs with LUVs after the incubation time. Different columns show (left) bright field, (middle) rhodamine dye, and (right) Cy5 signals.

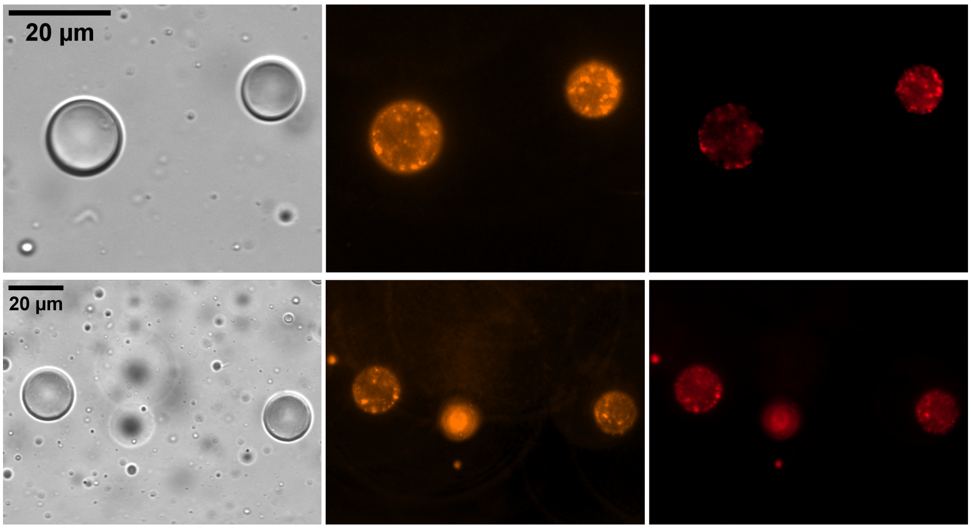

Figure S4: Additional representative images with the same experimental condition as those shown in Fig. 5 (bottom row) of the main text: Fusion behavior of DOPE and PLIN5 decorated LDs with LUVs after the incubation time. Different columns show (left) bright field, (middle) rhodamine dye, and (right) Cy5 signals.
